# Supplementary material for: Influence of Brewing Method on the Content of Selected Elements in Yerba Mate (Ilex paraguarensis) Infusions
Source: Foods. 2023 Mar 2;12(5):1072. doi: 10.3390/foods12051072 (PMC10001064; doi:10.3390/foods12051072)
Supplement: Supplementary file 1 [file foods-12-01072-s001.zip › foods-2235207-supplementary.pdf]

Supplementary Material

Influence of extraction parameters on the content of selected elements in yerba mate (Ilex paraguarensis) infusions

Jędrzej Proch, Anna Różewska, Aleksandra Orłowska, Przemysław Niedzielski*

Department of Analytical Chemistry, Faculty of Chemistry, Adam Mickiewicz University, Uniwersytetu Poznańskiego 8, 61–614 Poznań, Poland.

***Corresponding author**

mail: pnied@amu.edu.pl

phone: +48 61 829 1574

Table S1. The list of elements in fresh tap water (mg L^{-1}) and blank samples (BEC, blank equivalent concentration, mg kg^{-1}) of each procedure (A–D), which were determined above instrument quantification limits (IQLs) and method quantification limits (MQLs) respectively.

Element	IQL (mg L^{-1})	Fresh tap water (mg L^{-1})	MQL (mg kg^{-1})	BEC (mg kg^{-1})			
				A (tap water, RT)	B (DI water, RT)	C (tap water, 80°C)	D (DI water, 80°C)
Al	0.018	0.036	0.28	4.78	2.57	2.17	1.56
Ca	0.073	178	1.1	2870	31.6	2970	9.3
Cu	0.0019	0.0060	0.028	0.219	0.054	0.156	0.044
Fe	0.017	0.030	0.25	5.06	3.08	1.77	1.74
K	0.113	20.5	1.7	374	270	349	274
Mg	0.053	8.71	0.79	166	6.09	166	2.77
Mn	0.0080	<IQL	0.12	5.07	2.83	1.63	1.70
Na	0.087	29.3	1.3	548	36.8	526	14.5
Ni	0.0044	<IQL	0.066	0.197	0.347	0.120	0.084
P	0.035	<IQL	0.52	5.11	3.42	1.66	1.87
S	0.12	34.9	1.8	1380	21.2	1660	14.4
Sr	0.0027	0.328	0.040	5.51	0.078	5.59	<MQL
Zn	0.0051	<IQL	0.076	0.222	0.257	0.125	<MQL

IQL – instrument limit quantification (10 σ criterium); MQL – method limit quantification (including ultrasound–assisted extraction); BEC – blank equivalent concentration; RT – room temperature; DI – deionized (water);

Table S2. Performance parameters obtained with ICP OES for determination of selected elements in yerba mate.

Element	Emission line (nm)	Calibration range (mg kg^{-1})	MQL(total) (mg kg^{-1})	MQL(ext) (mg kg^{-1})
Al	396.152	MQL–50000	0.92	0.28
As	188.980	MQL–500	0.10	0.029
Ca	422.673	MQL–100000	3.8	1.1
Cd	214.439	MQL–1000	0.027	0.0081
Co	238.892	MQL–1000	0.12	0.037
Cr	267.716	MQL–1000	0.13	0.040
Cu	327.395	MQL–1000	0.093	0.028
Fe	238.204	MQL–12500	0.85	0.25
Hg	194.164	MQL–500	0.51	0.15
K	769.897	MQL–100000	5.8	1.7
Mg	285.213	MQL–100000	2.6	0.79
Mn	257.610	MQL–12500	0.41	0.12
Mo	202.032	MQL–500	0.12	0.037
Na	588.995	MQL–50000	4.2	1.3
Ni	231.604	MQL–1000	0.22	0.066
P	213.618	MQL–25000	1.7	0.52
Pb	220.353	MQL–1000	0.28	0.083
Rb	780.026	MQL–1000	0.57	0.17
S	181.972	MQL–25000	6.1	1.8
Se	196.026	MQL–500	0.62	0.19
Sr	460.733	MQL–1000	0.13	0.040
V	292.401	MQL–500	0.18	0.053
Zn	213.857	MQL–1000	0.25	0.076

MQL(total) – method quantification limit (microwave–assisted mineralization); MQL(ext) – method quantification limit (ultrasound–assisted extraction).

Table S3. The ICP OES analysis of certified reference materials (CRMs): hardwood biomass material (SRM 2790), tobacco leaves (OBTL–5, PVTL–6), mushroom powders (IPE–120, CS–M–3), prepared using the procedure of microwave–assisted acid mineralization. All results presented as mean±SD (n=3), except where is noted.

	**Inorganic Constituents in Hardwood Biomass Material (SRM® 2790, NIST, USA)**			**Oriental Basma Tobacco Leaves (OBTL–6, INCT, Poland)**			**Polish Virginia Tobacco Leaves (PVTL–6, INCT, Poland)**			**Mushroom (*Agaricus bisporus)* (IPE–120, WEPAL, Netherlands)**			**Microelements in Mushroom Powder (CS–M–3, INCT, Poland)**		
**Element**	**Certified (±U) ($mg\ kg^{-1}$)**	**Detected ($mg\ kg^{-1}$)**	**Recovery (%)**	**Certified (±U) ($mg\ kg^{-1}$)**	**Detected ($mg\ kg^{-1}$)**	**Recovery (%)**	**Certified (±U) ($mg\ kg^{-1}$)**	**Detected ($mg\ kg^{-1}$)**	**Recovery (%)**	**Certified (±U) ($mg\ kg^{-1}$)**	**Detected ($mg\ kg^{-1}$)**	**Recovery (%)**	**Certified (±U) ($mg\ kg^{-1}$)**	**Detected ($mg\ kg^{-1}$)**	**Recovery (%)**
Al	92.06±0.89[a]	85.1±4.1	93±5	1980±280	1930±77	98±4	252±49	248±7	98±3	x	16.5±0.5	–	x	1460±45	
As	0.0335±0.0089*	BQL	–	0.668±0.086	0.55±0.03	83±4	0.138±0.010	0.17±0.01	120±6	0.137±0.067[b]	BQL	–	0.651±0.026[a]	0.71±0.05	109±8
Ca	x	2400±149	–	39960±1420	46400±2830	116±7	22970±780	25000±1650	109±7	393±106[b]	453±23	115±6	x	1040±53	
Cd	x	0.057±0.002	–	2.64±0.14	2.25±0.05	85±2	2.23±0.12	2.18±0.08	98±4	0.0772±0.0192	0.062±0.001	80±1	1.229±0.11[a]	1.12±0.01	91±1
Co	0.098±0.012*	BQL	–	0.981±0.067	0.83±0.07	85±7	0.154±0.007	0.13±0.01	82±3	0.0133±0.0053[b]*	BQL	–	x	0.194±0.01	
Cr	1.590±0.150	1.35±0.06	85±3	6.3[c]	5.28±0.17	84±3	0.911[c]	0.75±0.04	82±4	0.5430±0.2463[b]	0.44±0.01	81±1	5.79±0.80[a]	5.65±0.09	98±2
Cu	1.545±0.030[a]	1.69±0.14	109±9	10.1±0.4	10.4±0.5	103±5	5.12±0.2	4.62±0.12	90±2	17.4±1.4	14.8±0.1	85±1	18.73±0.70[a]	20.0±0.2	107±1
Fe	82.9±5.5	83.2±8.3	100±10	1490[c]	1420±77	96±5	258[c]	235±4	91±2	31.4±8.1[b]	25.5±0.4	81±1	x	1050±16	
Hg	0.01323±0.0002*	BQL	–	0.0209±0.0013*	BQL	–	0.0232±0.0016*	BQL	–	0.0612±0.0040[b]*	BQL	–	2.849±0.104[a]	2.66±0.06	94±2
K	1040±130	1210±98	116±9	22710±760	22300±1070	98±5	26400±900	28700±774	109±3	43600±2360	40300±604	92±1	x	21500±322	
Mg	189±11	158±14	84±7	8530±340	8090±461	95±5	2410±90	2410±29	100±1	1130±86	937±24	83±2	x	536±14	
Mn	52.4±1.9	59.0±4.5	113±9	180±6	156±5	87±3	136±5	136±4	100±3	5.3±1.2	5.1±0.3	96±5	x	46.5±2.5	
Mo	x	1.24±0.06	–	0.414±0.062	0.34±0.03	89±6	0.396±0.029	0.34±0.03	87±7	0.179±0.030[b]	0.20±0.01	109±8	x	0.18±0.01	
Na	26.0±5.3	23.1±2.1	89±8	435[c]	378±22	87±5	62.4[c]	72.0±4.4	115±7	577±79	538±8	93±1	x	77.5±1.1	
Ni	x	0.88±0.02	–	8.5±0.49	7.38±0.22	87±3	1.49±0.14	1.20±0.02	81±1	0.246[c]	0.27±0.01	111±3	x	3.38±0.10	
P	x	62.5±2.8	–	1700±120	1480±53	87±3	2420±150	2470±79	102±3	9820±631	8970±117	91±	x	3850±50	
Pb	x	2.44±0.12	–	2.01±0.31	2.12±0.10	106±5	0.972±0.147	1.10±0.05	113±5	0.188[c]*	BQL	–	1.863±0.108[a]	1.89±0.05	101±2
Rb	3.110±0.099[a]	3.18±0.15	102±5	19.1±1.0	20.9±0.7	109±4	5.97±0.28	7.32±0.31	123±5	14.1[c]	12.7±0.6	90±5	x	147±7	
S	x	1110±53	–	4550±910	4200±176	92±4	3780±590	4220±127	112±3	2100±161	2090±38	100±2	x	13600±244	
Se	0.0102±0.0023*	BQL	–	x	BQL	–	x	BQL	–	0.729[c]	0.69±0.06	94±8	17.43±1.36[a]	14.5±1.2	83±7
Sr	x	9.83±0.57	–	105±5	121±4	115±4	133±6	136±8	103±6	0.76[c]	0.79±0.05	103±6	x	1.85±0.11	
V	0.243±0.011	0.23±0.01	95±4	4.12±0.55	3.02±0.13	73±3	0.405±0.056	0.41±0.04	100±10	0.07[c]*	BQL	–	x	2.78±0.13	
Zn	9.26±0.9	8.51±0.61	92±5	52.4±1.8	43.5±1.3	83±2	43.6±1.4	41.4±1.9	95±4	42.2±4.2	33.7±0.5	80±1	113.3±3.3[a]	97.0±1.5	86±1

U – expanded uncertainty of certified value; SD – standard deviation; [a] – reference value; [b] – indicative value; [c] – informative value; BQL—below (method) quantification limit; * – value below MQL; x – not certified value.

Table S4. The comparison of ultrasound–assisted extraction (USN) and conventional brewing (CON) for all procedures (A–D) and yerba mate samples (n=7). All results were given as the relative percentage (%) of extractable contents (USN to CON).

**Procedure**	**A (tap water, RT)**		**B (DI water, RT)**		**C (tap water, 80ºC)**		**D (DI water, 80ºC)**	
**Elements**	**Median (min–max)**	**AQL (USN–CON)**	**Median (min–max)**	**AQL (USN–CON)**	**Median (min–max)**	**AQL (USN–CON)**	**Median (min–max)**	**AQL (USN–CON)**
Al	118 (99–139)	7–7	103 (75–164)	7–7	105 (62–137)	7–7	140 (105–191)	7–7
As	127 (78–144)	7–6	138 (50–218)	7–7	112 (38–164)	7–5	149* (129–170)	7–2
Ca	ND	ND	106 (92–133)	7–7	ND	ND	143 (107–182)	7–7
Cd	ND	0	ND (only USN)	3–0	43^{USN}	4–1	ND (only USN)	3–0
Co	152 (115–197)	6–5	106 (95–148)	6–6	104 (62–158)	6–6	159 (110–212)	6–6
Cr	148 (108–174)	5–5	114 (91–311)	5–5	125 (64–149)	5–5	153 (119–236)	5–5
Cu	116 (79–140)	7–7	97 (77–152)	7–7	104 (49–131)	7–7	154 (115–213)	7–7
Fe	ND	0	ND (only USN)	7–0	94 (21–189)	7–6	199 (107–471)	7–6
K	116 (76–145)	7–7	100 (81–164)	7–7	97 (57–135)	7–7	150 (109–205)	7–7
Mg	115 (71–146)	7–7	97 (87–163)	7–7	114 (58–142)	7–7	160 (118–222)	7–7
Mn	123 (111–149)	7–7	101 (93–158)	7–7	120 (64–138)	7–7	151 (118–213)	7–7
Na	ND	ND	237 (138–360)	7–7	ND	ND	99 (66–225)	7–7
Ni	119 (102–166)	7–7	117 (108–191)	7–6	117 (65–147)	7–7	144 (110–204)	7–7
P	123 (117–151)	7–7	98 (87–178)	7–7	111 (64–143)	7–7	153 (127–193)	7–7
Rb	127 (116–158)	7–7	101 (77–176)	7–7	99 (52–151)	7–7	174 (120–246)	7–7
S	ND	ND	111 (88–233)	7–7	ND	ND	154 (118–224)	7–7
Se	ND	0	ND (only USN)	1–0	ND	0	99^{USN}	3–1
Sr	147^{USN}	2–1	99 (92–143)	7–7	69^{CON}	1–2	156 (109–206)	7–7
Zn	124 (109–163)	7–7	105 (88–170)	7–7	115 (62–143)	7–7	143 (114–195)	7–7

RT – room temperature; DI – deionized (water); ND – no data; ^{USN} – single value of the USN/CON ratio with the dominance of USN results; ^{CON} – single value of the USN/CON ratio with the dominance of CON results; * – mean of the USN/CON ratios (if n = 2) ,

Table S5. The comparison of ultrasound–assisted extraction in boiling water (DI water, 100ºC) with procedure D (DI water, 80ºC) for selected samples of yerba mate (n=3). All results were given as the relative percentage (%) of extractable content (100ºC to 80ºC).

Element	Mean±SD	Median (min–max)
**Al**	126±17	132 (103–142)
**As**	163±22	157 (140–193)
**Ca**	100±10	101 (87–111)
**Cd**	95±14	103 (75–106)
**Co**	132±13	128 (119–150)
**Cr**	128±18	126 (106–151)
**Cu**	140±17	144 (117–159)
**Fe**	138±20	142 (112–160)
**K**	146±14	154 (126–158)
**Mg**	153±18	157 (129–173)
**Mn**	135±16	138 (113–153)
**Na**	184±72	137 (129–286)
**Ni**	135±16	140 (114–152)
**P**	136±15	138 (117–153)
**Rb**	159±18	169 (133–174)
**S**	113±13	113 (97–128)
**Se**	132±11	125 (124–148)
**Sr**	115±9	110 (107–128)
**Zn**	122±20	123 (96–146)
